# How accurate is the visual estimation of bowel length by endoscopic surgeons?

**DOI:** 10.3389/fsurg.2022.1001329

**Published:** 2022-11-07

**Authors:** Sahar Mirzaee, Mahdieh Golzarand, Reza Parsaei, Karamollah Toolabi, Alireza Amirbeigi

**Affiliations:** ^1^ Department of Surgery, Tehran University of Medical Sciences, Tehran, Iran; ^2^ Nutrition and Endocrine Research Center, Research Institute for Endocrine Sciences, Shahid Beheshti University of Medical Sciences, Tehran, Iran; ^3^ Department of Surgery, West Nikan Hospital, Tehran, Iran; ^4^ Clinical Research Development Unit, Shahid Bahonar Hospital, Kerman University of Medical Sciences, Kerman, Iran

**Keywords:** bariatric surgery, gastric bypass, limb length, bowel measurement, intestine

## Abstract

**Background:**

Measurement of small bowel length is an essential step in performing bariatric surgery. Surgeons need to measure bowel length in order to create alimentary and biliopancreatic limbs. Inaccurate bowel measurement may affect the outcome of surgery. However, it is not clear how accurate the measurement of bowel length is by surgeons.

**Methods:**

Two image quizzes marking certain lengths of jejunum were sent to participants. They were asked to estimate the length of marked bowels in maze quizzes. The Error of estimation, prevalence of significant error (error greater than 30 percent of actual length), and the relationship between different participant characteristics was investigated.

**Results:**

A total of 86 participants answered the questionnaire. The mean error of estimation was 4.62 cm (27%). Twenty-eight participants (33%) had significant errors in estimation of bowel length.

**Conclusion:**

While there are surgeons that can estimate bowel length with decent accuracy, significant errors in estimation of bowel length are not uncommon among surgeons. Surgeons should consider adopting techniques for accurate measurement of the small intestine.

## Introduction

Measurement of small bowel length is an essential step in performing some bariatric surgeries. Surgeons need to measure bowel length in order to create alimentary and biliopancreatic limbs. The length of the bypassed bowel can affect the outcome of surgery. A long limb may be associated with excessive weight loss or micronutrient deficiency (1, 2). A short limb may lead to inadequate weight loss and failure of surgery (3). Accurate measurement of distances is also important in oncologic surgery, where an adequate margin of resection is imperative for curative surgical treatment. However, it is not clear how accurate the measurement of bowel length by endoscopic surgeons is. Using rulers and other measuring tools is not easy in laparoscopic surgery and is not a common practice in many centers. Surgeons mostly use their visual estimation of distances to measure bowel length. Two-dimensional vision in laparoscopic surgery and a lack of depth perception impair the precision of the surgeons' visual estimation of distances. Although three dimensional vision is provided in robotic surgery consoles, its impact on accuracy of distance estimation is not completely understood. There is evidence that the accuracy of measurement of bowel length can vary among different surgeons and may not be as precise as expected (4). We carried out this study to assess the accuracy of bowel length estimation by endoscopic surgeons.

## Methods

### Participants

Members of the Iranian endoscopic surgery society were enrolled in the study. We contacted them by phone and explained to them our study. Then the link to a web-based questionnaire was sent to them *via* e-mail or social media applications. In the questionnaire, we asked about general characteristics of participants, including age, gender, years of surgical experience, whether they perform bariatric surgeries, and the average weekly volume of their bariatric surgeries. Finally, we wanted participants to estimate the marked length of the bowel between two graspers shown in two image quizzes.

### Image quiz

**Figure 1:**
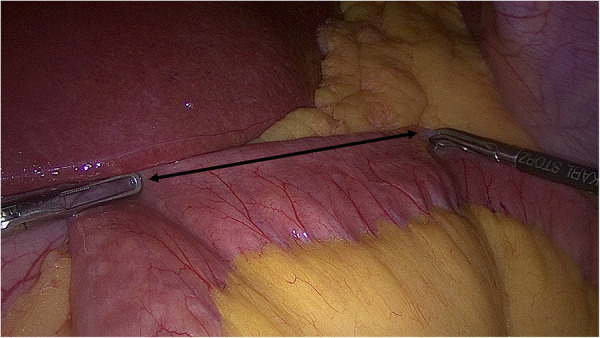
Image quiz marking 8 cm of jejunum was sent to participants.

During a laparoscopic Roux-en-Y gastric bypass (LRYGB) surgery, a paper ruler was introduced into the abdomen. Using the ruler for precise measurement, we held 8 cm of jejunum between two graspers and took a photo of that. The specified length of jejunum between two graspers was marked in the photo (Figure 1). Using the same method, another photo was taken during another LRYGB, assigning 9 cm of jejunum. The two photos were included in a web-based questionnaire that was sent to the participants. The lengths of 8 cm and 9 cm were acquired through a random number generator that was set to produce numbers from 5 to 15.

### Statistical analysis

The error of bowel length estimation was calculated as the absolute value of the sum of bowel length estimation in two image quizzes minus 17 (the actual length of two quizzes). A significant error was defined as one that was greater than 30% of the actual length. The Kolmogorov-Smirnov test was used to determine the normality of continuous variables. Continuous variables were presented as the mean ± standard deviation. Categorical variables were shown as a number and a percentage. The mean error of length estimation in different participants' subgroups was compared using the Mann-Whitney U test. Differences in the prevalence of significant errors in various participant subgroups were assessed using chi-square or Fisher's exact test as indicated. The IBM SPSS statistics for Windows version 22 (IBM Corporation, Armonk, NY) was used for analysis.

## Results

A total of 86 members of the Iranian endoscopic surgery society participated in the study. The general characteristics of participants are shown in Table 1. The mean error of estimation was 4.62 cm (27%). Twenty-eight participants (33%) had significant errors in estimation of bowel length. An error greater than 25% was observed in 39 (45%) of the participants. No association was found between errors of estimation and different characteristics of participants, including age, gender, years of surgical experience, and volume of surgeries. The relationship between error and different participants' subgroups is shown in Table 2.

**Table 1:** General characteristics of participants.

Characteristics	Mean ± SD^a^
Age (years)	45.8 ± 8.45
Age subgroups	
* <*45 *years*	44 (51%)
* ≥*45 *years*	42 (49%)
Gender	
* Female*	4 (5%)
* Male*	82 (95%)
Years of surgical experience	12.1 ± 8.8
Surgical experience subgroups	
* <*10 *years*	48 (56%)
* ≥*10 *years*	38 (44%)
Bariatric surgeon	
* Yes*	58 (67%)
* No*	28 (33%)
Volume of bariatric surgery per week	6.4 ± 5.7
Volume of bariatric surgery subgroups	
* <*4 *per week*	30 (52%)
* ≥*4 *per week*	28 (48%)
Total number	86

^a^ Non-continuous variables were expressed as count (percent).

**Table 2:** Relationship between different participants’ characteristics and error of bowel length estimation.

Participants characteristics	Mean error (CM)^a^	*P* value	Significant error^b^		*P* value
			Yes	No	
Age					
<45 years	4.7 (28%) ± 4.6	0.87	13 (30%)	31 (70%)	0.70
≥45 years	4.6 (27%) ± 3.9		15 (36%)	27 (64%)	
Gender					
Female	6.2 (36%) ± 5.0	0.49	2 (50%)	2 (50%)	0.39
Male	4.8 (28%) ± 3.6		26 (32%)	56 (68%)	
Years of surgical experience					
<10 years	4.7 (28%) ± 4.4	0.64	15 (31%)	33 (69%)	0.95
≥10 years	4.5 (26%) ± 4.1		13 (34%)	25 (66%)	
Bariatric surgeon					
Yes	4.0 (24%) ± 2.7	0.30	18 (31%)	40 (69%)	0.30
No	5.9 (35%) ± 6.3		10 (36%)	18 (64%)	
Volume of bariatric surgery					
<4 per week	4.5 (26%) ± 3.1	0.28	10 (33%)	20 (67%)	0.30
≥4 per week	3.5 (21%) ± 2.2		8 (29%)	20 (71%)	
Total	4.6 (27%) ± 4.3		28 (33%)	58 (67%)	

^a^ Numbers in parentheses shows percent error.

^b^ Errors greater than 30% of actual bowel length were considered as significant error.

## Discussion

Significant errors in visual estimation of bowel length were not uncommon in our population. Previous studies have shown that human distance perception is generally inaccurate (5). Norman et al. (6) carried out an experiment to assess the accuracy of distance ratio estimation (how large one environmental distance is relative to another) in an indoor physical space. They found that 10% of observers overestimated distance ratios while 50% underestimated them. The distance ratio is especially important in endoscopic surgery. Some surgeons measure a distance by estimating the ratio of that distance to a known object size. The known object size could be a grasper head size, a marked instrument shaft, or the width of a laparoscopic instrument shaft, which is usually 5 mm. Inaccurate distance estimation has also been reported in other studies that investigated distance measurements in surgical settings. Lussenden et al. (7) enrolled 10 surgical residents and 4 attending surgeons in their study. They placed a 500 cm porcine intestine sample in a laparoscopy trainer box and told participants to measure 100 cm of intestine. The average error was 24 cm for both residents and attending surgeons. Gazer et al. (4) asked 14 participants to measure different lengths of bowel in a porcine *in vivo* model. They found that assessment of bowel length by participants was inaccurate in laparoscopy. Some investigators used string or rope to assess the accuracy of measurement in participants. Isreb et al. (8) recruited 22 surgeons to measure 150 cm of a string placed in the laparoscopy training box. The mean length of the measured string was 115.4 cm. Due to two-dimensional vision and loss of depth perception, estimation of distances in laparoscopic settings may be less accurate compared to open or robotic surgery with three dimensional vision. However, even in open surgery, measurement of bowel length may not be precise. Muise et al. (9) investigated the accuracy of 12 participants in measuring bowel length in euthanized rabbits. They found great variations in the measurement of bowel length among surgeons. They also concluded that measurement by tape has superior accuracy compared to marked graspers. The design of our study and sending image quizzes to surgeons allowed us to enroll more participants compared to similar studies. Besides, all of our participants were practicing surgeons with experience in endoscopic surgery. Although there were surgeons that could accurately assess the distances in a laparoscopy picture, our finding is consistent with the results of other studies that show estimation of bowel length is prone to error and inaccuracy. Some studies have found that older adults can estimate distances more accurately compared to younger adults (10). In our study, the range of age distribution was narrow and the average age in two participant subgroups was similar, so a significant difference in accuracy was not observed. There are studies that have reported that distance perception was more accurate in male participants compared to females (11). The number of female participants was small in our study. So, we cannot reach a conclusion about the effect of gender on distance estimation accuracy based on our findings. We did not find any relationship between the volume of surgery and the accuracy of distance perception. Although volume of surgery is a good indicator of experience, higher volume may not necessarily correlate with surgical skill and competency. Besides, the volume of surgery in our study is self-reported and may not be accurate. It also reports the current weekly volume of surgery and does not show the cumulative experience of participants.

The error in measuring bowel length was greater than 30% in 28 (33%) of our participants. The clinical implications and consequences of such a percentage of errors are not completely clear. Current evidence regarding the clinical outcome of different alimentary, biliopancreatic, and common channel limbs is conflicting. Khalaj et al. (12) compared the outcomes of different lengths of biliopancreatic limbs in patients who underwent one anastomosis gastric bypass (OAGB). They reported that OAGB with a 200 cm biliopancreatic limb was associated with a higher rate of protein-calorie malnutrition compared to a 160 cm biliopancreatic limb. Slagter et al. (13) found that attained BMI was higher in OAGB patients with a 200 cm biliopancreatic limb compared to those with a 150 cm or 180 cm biliopancreatic limb. Shah et al. (14) investigated the outcome of LRYGB based on the total length of the alimentary and biliopancreatic limbs. They reported that a total of 310 cm of bypassed intestine delivered more weight loss compared to the 260 cm and 210 cm subgroups. According to these studies, a 30 percent deviation from the intended length of the bypassed intestine can result in an unfavorable outcome or additional complications. Nabil et al. (15) reported that bypassing more than 200 cm of small bowel in OABG predisposes patients to nutritional deficiency. Gan et al. (16) carried out a meta-analysis to address the influence of the alimentary limb on the outcome of LRYGB. They reported that an alimentary limb of 130 cm to 150 cm produced superior results compared to an alimentary limb of 40 cm to 100 cm. Mahawar et al. (3) published a systematic review regarding the effect of the small bowel limb on the outcome of LRYGB. They recommended that the total length of the alimentary and biliopancreatic limbs should be between 100 cm and 200 cm for best results. On the other hand, Ahmed et al. (17) found no difference in the outcome of LRYBG in various alimentary limbs between 50 cm and 100 cm. They also reported similar results in patients with different common channel limbs, ranging from 320 cm to 520 cm. Boyle et al. (18) investigated the relationship between weight loss and the length of the biliopancreatic limb in OAGB surgery. No difference in weight loss was found between 150 cm and 200 cm biliopancreatic limbs.

A potential confounding factor in investigating the impact of limb length on the outcome of surgery is the inaccuracy of bowel length measurement in clinical studies. The accuracy of bowel measurement cannot be figured out in all studies. For instance, in the current study, seven original research papers have been referred to about the relationship between limb length and the result of surgery. The method of measurement was not disclosed in five studies (3, 12–14, 17). In one study, a rubber band was used for measurement (15). This method is believed to be the most accurate method of measurement. In one study, a marked grasper shaft was used to aid measurement. Previous studies have shown that estimation of length by comparing the ratio of the desired distance to a known object can be erroneous (6, 9). Inaccurate bowel length measurement may affect the result of studies about the relationship between different limb lengths and the outcome of surgery.

A limitation of our study is that we only assessed the accuracy of length estimation in our participants. However, it should be noted that visual assessment of distances is not the only skill needed to measure bowel length during surgery. Proper manipulation of the bowel and avoiding overstretching of the bowel is essential in bowel measurement. Because of the intestine's flexibility and the tethering effect of the mesentery, some bowel stretching may happen. So the actual error in bowel measurement in surgery may be higher than our results.

Despite the controversies and inconsistencies in literature, the adverse outcomes of inappropriately long or short bowel limbs remain a concern. If the surgeon is too inaccurate in measuring the small intestine, malnutrition, vitamin deficiency, or inadequate weight loss is a potential consequence.

## Conclusion

Although there are surgeons that can estimate bowel length with decent accuracy, significant error in visual estimation of distances is not uncommon among surgeons. Surgeons would consider adopting techniques for accurate measurement of the small intestine. According to previous studies [9], measuring bowel length by estimating the ratio of bowel to a known independent object like the grasper head may not be accurate. Direct measurement with a tape or paper ruler is the preferred method.

## Data Availability

The original contributions presented in the study are included in the article/Supplementary Material, further inquiries can be directed to the corresponding author/s.
